# Classifying interpersonal synchronization states using a data-driven approach: implications for social interaction understanding

**DOI:** 10.1038/s41598-023-37316-5

**Published:** 2023-07-10

**Authors:** Roi Yozevitch, Anat Dahan, Talia Seada, Daniel Appel, Hila Gvirts

**Affiliations:** 1 Department of Computer Science, Ariel University, Ariel, 40700 Israel; 2 Department of Software Engineering, Braude College of Engineering, Karmiel, 216100 Israel; 3 Department of Behavioral Sciences, Ariel University, Ariel, 40700 Israel

**Keywords:** Cooperation, Empathy

## Abstract

This study presents a data-driven approach to identifying interpersonal motor synchrony states by analyzing hand movements captured from a 3*D* depth camera. Utilizing a single frame from the experiment, an XGBoost machine learning model was employed to differentiate between spontaneous and intentional synchrony modes with nearly $90\%$ accuracy. Our findings demonstrate a consistent pattern across subjects, revealing that movement velocity tends to be slower in synchrony modes. These insights support the notion that the relationship between velocity and synchrony is influenced by the cognitive load required for the task, with slower movements leading to higher synchrony in tasks demanding higher cognitive load. This work not only contributes to the limited literature on algorithms for identifying interpersonal synchrony but also has potential implications for developing new metrics to assess real-time human social interactions, understanding social interaction, and diagnosing and developing treatment strategies for social deficits associated with conditions such as Autism Spectrum Disorder.

## Introduction

Interpersonal Synchrony (IS) is defined as ”the dynamic and reciprocal adaptation of the temporal structure of behavior between interactive partners”^1^. In simple words, interpersonal synchrony refers to the matching of behaviors, movements, and gestures between two or more people. Mirroring the body language of the person you’re talking to is a good example of the phenomenon. Interpersonal synchrony can create a feeling of connection and understanding between people and is often seen in close relationships such as romantic partnerships or friendships. It can also be used intentionally, for example, by a therapist, a salesperson, or when people follow instructions and possibly follow an external rhythm as in drumming or dancing^2^.

The importance of interpersonal synchrony in social interactions cannot be overstated, as it plays a vital role in the development of infants^3,4^, the formation of affiliative bonds^5^, the encouragement of prosocial behaviors^6^, and the recognition of emotions^7^. In group settings, IS contributes to cohesion and overall performance^8^. Both spontaneous and intentional synchrony are essential aspects of social behavior and have been linked to reward, motivation, and collaboration^9^.

A notable framework for measuring synchronization is the mirror game where players mimic each other’s movements. Participants can either move alone, move together with no specific instruction, or be instructed to in synchronization. Variations of this game have been applied in several studies using different types of recording devices. The evaluation of synchrony is usually quantified by extracting a time series that represents the movement of each participant and applying a measure of coherence or similarity between the two-time series.

Despite the wealth of research acknowledging the vital role of IS in social interaction, there is a notable gap in the literature regarding the application of machine learning (ML) algorithms to classify and understand various types of interpersonal synchrony. Current studies predominantly focus on ML application in participant-type classification, such as between individuals with and without Autistic Spectrum Disorder (ASD), rather than the specific synchronization states. In addition, these studies often utilize time series data, offering limited insights into the individual characteristics of various synchrony states (see section "Machine learning for classification of synchronization" for details).

This work utilizes a dedicated 3*D* hand gesture device to capture each hand’s exact posture and velocity vectors (see section "The leap motion controller"). We apply ML algorithms to learn the different properties to distinguish between three synchronization states—Autonomous, Spontaneous, and Intentional synchrony. Unlike previous methods for evaluating synchronization that compares time series measurements, we base the classification on a single snap-shot of the experiment. The ability to classify synchronization states based on a single snapshot of the experiment marks a significant stride in IS research.

Crucially, the application of ML not only facilitates the classification of synchronization states but also provides a *data-driven approach* that offers valuable insights into the dynamics of the interaction. The features utilized for this model disclose a consistent pattern among subjects, wherein the movement velocity is typically slower in states of synchrony. This pattern tells an interesting story about the relationship between position and velocity in these states, thereby enhancing our existing comprehension of interpersonal synchrony. We discuss these insights in section "Discussion".

The implications of this novel approach extend beyond advancing our understanding of IS. It opens up new avenues for researching and understanding the nature of social interactions, the cues we subconsciously use and respond to, and how these dynamics may be harnessed for therapeutic and practical purposes.

The structure of this paper is organized as follows: Section "Related works" provides an overview of prior work in the field. Section "The leap motion controller" introduces the recording device used in the study. Section "Our mirror game experiment" outlines the experimental setup. Section "Machine learning utilization" details the ML algorithms and features employed. Sections "Results" and "Discussion" present the results and discussion, respectively. Finally, Section "Conclusions and future work" concludes the paper with a summary of the key findings and suggestions for future research.

## Related works

### The mirror game

As mentioned above, the Mirror Game is a common mimicry exercise where players mimic each other’s full-body movements. It is often used In theater, dance, and movement therapy^10^. It is one of the first game methodologies that was developed for research purposes in order to measure synchronization and states of togetherness. In the original mirror game, experimental setting^11,12^ two players faced each other holding handles that can move along parallel tracks. The players were instructed to move together in a synchronized and interesting manner. According to each player’s velocity profile, the players’ synchronization was calculated according to the mean relative difference in velocity and the timing differences between zero-velocity events.

In the following years, many studies extended this paradigm and measured movement using different technologies.

One work, for example, extended the original mirror game methodology of one-dimensional movement of moving handles to 3*D* movement^12^. Other recording technologies include video recordings, Kinect depth cameras, 3*D* cameras, and sensors as wrist work accelerometers^13–15^.

### Synchronization metrics

Is there an objective (mathematical) measure of synchronization? Since there are different interpretations of the term, several methods have been applied to evaluate a synchronization metric. The most common approach to evaluate synchronization is by assessing the correlation between two-time series measurements of a dyad members’ movements.

A time series can represent a series of sampling of different aspects of a movement. Some studies obtain a time series describing the velocity of each participant according to a sampling of a marker attached to the body or hands of participants^16^, or to the movement of a handle^11^. A time series that evaluates movement can also be obtained by an accelerometer attached to the body and or head^17,18^, and sampling from the acceleration data of each participant. In tasks of tapping, two participants are instructed to tap in synchrony. A time series for each participant can be obtained by measurements of the interval between taps (Intertap- intervals)^19^. A recent study has recently proposed novel measure, known as dynamic pose similarity (DPS)^2^. This measure creates a time series for each participant that aggregates the position of fifteen joints with the directions of movement. When using methodologies of videotaping of participants, individual body movement is assessed by the frame-difference method of motion energy analysis (MEA)^20,21^. For each consecutive pair of video frames, the algorithm counts the number of pixels in which the grey intensity changed between. This results in a time series of motion intensity values for each participant.

One prevalent time series analysis is the Rolling-Window-Time-Lagged-Cross-Correlation (RWTLCC)^22^. RWTLCC provides correlations between two data streams across different time lags. This method considers that the time series are not necessarily perfectly aligned in time. Therefore the RWTLCC calculates correlations for each of a range of possible lags. Other methods that have been suggested to evaluate the synchrony of time series include Pearson product-moment correlations^7^, dynamic time warping^23^, and phase synchrony^24^. An examination of different methods of linear time series analysis, on time series extracted by Motion energy analysis of videotapes of moving dyads (^25^) concluded that each method measures different aspects of synchrony, such as the strength of synchrony of the total interaction vs during synchronization intervals, and the strength vs the frequency of synchrony.

While the measures described above aim to quantify the level of synchronization, other approaches try to classify the state of synchrony. Identifying whether participants are synchronized can be valuable in real-life therapeutic settings. One work measured children’s social engagement in a group that included autistic children participating in a theatrical workshop by measuring interpersonal movement synchrony^18^. They used wrist-worn accelerometers to measure non-verbal social coordination within the group. Synchronization was calculated using a cross-wavelet similarity comparison between participants’ movement data. For tasks of classification, machine learning can offer valuable techniques and insights.

### Machine learning for classification of synchronization

IS data is very rich and varied. Thus, it may be analyzed in terms of rhythm, in terms of location, form, etc. For such data, it is often useful to harness ML algorithms. These algorithms have become the most relevant methodology for classifying data without making assumptions about the nature of the data in a bottom-up data-driven approach^26^. Motor behavior is a visible output of complex internal intentions. In recent years, advances in deep learning techniques have enabled scientists to study patterns of motor behavior with high accuracy^27^. Such techniques involve using ‘skeletonized’ data and 3D shapes (how ‘skeletonized’ data and 3D shapes) evolve as a time series. These series can be interpreted as different poses in the process of pose estimation using deep learning techniques^28–31^. The authors of^32^ have used the Open Pose deep learning-based 2D pose estimator o quantify movement-music synchrony. Some studies have used machine learning methods to classify patterns of IS. A proof of concept study showed that interpersonal coordination movement patterns of TD vs ASD dyads can be classified using machine learning methods^33^. Intra-personal Synchrony between the head and upper body was quantified using Motion Energy Analysis, and the resulting time series were used as features for classification using a support vector machine with a linear kernel algorithm. In another work, measures of IS that were extracted from naturalistic video recordings were used to classify participants as belonging to ASD or control groups^34^. A time series of facial expression features were extracted using the Openface algorithm (A python and Torch implementation of face recognition with deep neural networks^35^), which extracts action units and three head pose parameters (pitch, yaw, roll). Head and body movement was assessed using a Motion Energy Algorithm (MEA) algorithm^20^. To assess IS, cross-correlation measures were applied. Several summary scores (e.g., mean, median, etc.) were used as features for a Support Vector Machine (SVM) classifier to classify dyads belonging to a mixed (ASD-TD) or non-autistic control (TD-TD) dyad. ^36^ proposed a data-driven approach to quantify vocal and linguistic synchrony. Vocal synchrony was assessed by extracting spectral features and measuring distances. Linguistic synchronization was assessed by measuring the lexical distance between sentences. These distances were used as features for classification using a linear and nonlinear SVM classifier and a linear regression model.

There are still only a few studies that harness Machine Learning to assess IS. To the best of our knowledge, there are no studies that use ML to classify between different types of synchronization, but rather all are used to classify between types of participants. Interestingly, while all approaches use different features, all features evaluate IS using a time series calculation of measures recorded from the two-time series of the dyad participants.

## The leap motion controller

**Figure 1:**
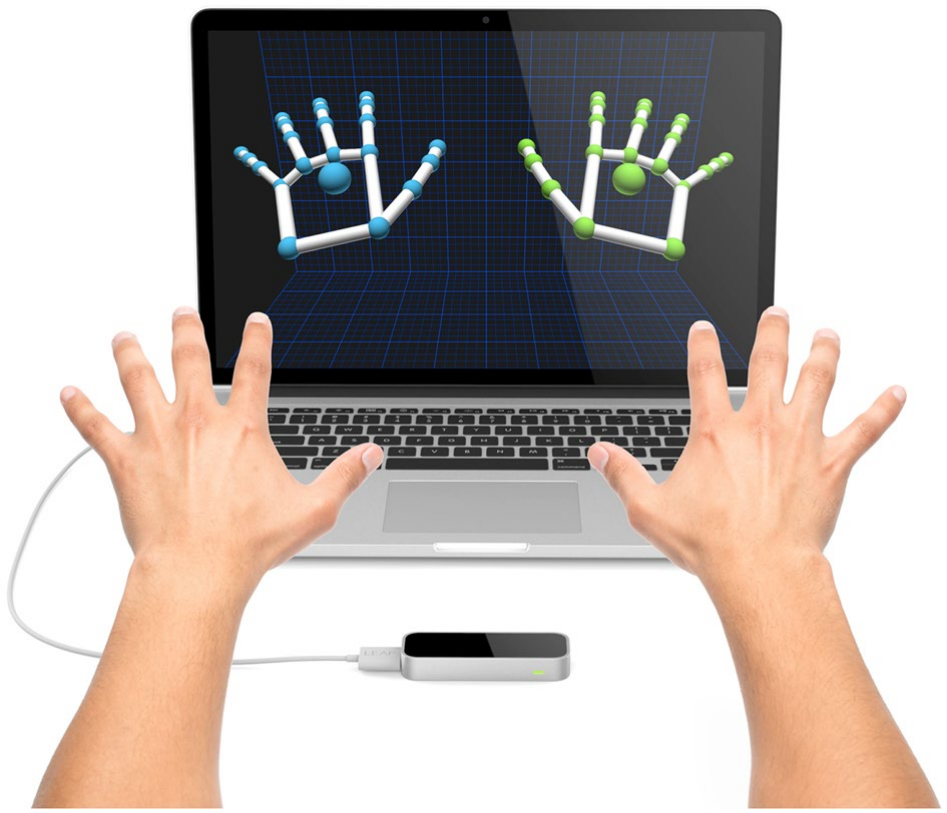
The leap motion controller (LMC) device.

Hand gesture recognition^37^ is attracting a growing interest due to its applications in many different fields, such as human-computer interaction, robotics, computer gaming, automatic sign-language interpretation, and more. The Leap Motion controller (LMC)^38^ device has opened new opportunities for gesture recognition. Differently from the Microsoft Kinect^39^, the LMC is explicitly targeted to hand gesture recognition and directly computes the position of the fingertips and the hand orientation. Although the amount of information is limited compared to other depth cameras (e.g., Kinect), the extracted data is more accurate with sub-millimeter accuracy. In contrast to standard multi-touch solutions, this above-surface sensor is designed for use in realistic stereo 3*D* interaction systems^40^. Figure 1 shows both the controller and a 3*D* output of two hands simultaneously.

The LMC is explicitly targeted to hand gesture recognition and directly computes the fingers’ position/velocity vectors.

## Our mirror game experiment

Twelve participants were recruited (ages 19-30). All participants were right-handed and were native speakers of Hebrew. The ethics committee of Ariel University approved the study protocol in accordance with the ethics approval guidelines of the University’s Ethics Committee. Informed consent was received from all participants. The data-set generated and analyzed during the current study is available in a public GitHub repository^41^.

As explained in the introduction, we measure the interpersonal synchrony of two people by inspecting hand gestures and measuring their similarity. Hand gestures are ecological and can occur naturally in a synchronization task.

The experiment was conducted as follows: two participants sat facing each other at two sides of a table. They were told to move in three different alignment states. These states are:

1. an Alone mode.
2. a Spontaneous mode
3. an Intentional Synchronization mode.

In the first state, each participant, in their turn, was asked to move their hand freely over the LMC. We denote this as an ”**Alone**” mode. The second state is very similar to the first. The only difference is that the two participants can see each other’s movement during the experiment. We denote this as an ”**Spontaneous**” state since spontaneous synchrony may occur in such states(see section 1).

**Figure 2:**
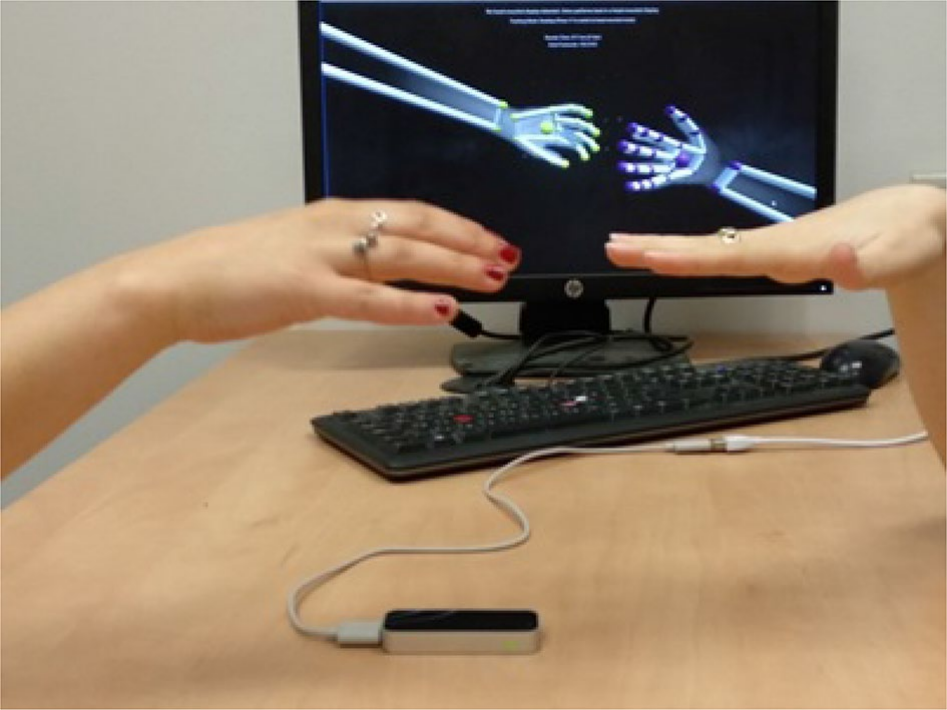
A picture from the experiment. Two persons sit facing each other and move their hands freely over the Leap Motion controller.

The last state is different. In the last state, two participants are asked to move *in synchronization*. They are invited to interpret ”synchronization” as they understand the term. We denote this state as ”**synchronization**” state. A picture from the experiment can be seen in Fig. 2.

The LMC reports the exact posture of each hand at $\approx 100 Hz$. The data can be extracted via the official *SDK*. A Python logger was implemented to save this data for further analysis.

The conducted experiment included the following three stages, in each of which a 75-second period was captured by the Leap Motion Controller at 120 *FPS* and exported into an Excel file containing all the captured information. The question we want to raise is twofold. **First**, given a single snap-shot of the mirror game, can one distinguish between ”alone”, ”spontaneous” and ”synchrony” modes? **Second**, based on the collected data of these three states, what observations can be made? We address both questions in the upcoming sections.

## Machine learning utilization

ML techniques have shown great results in classification and/or prediction problems in numerous fields. In this section, we harness those tools in the field of IS. There is a famous quote by scholar Andrew Ng that any task a person can do (compute) in one second of thought, an AI system can also achieve^42^. Empirical observation has taught us that while it is relatively an easy task to distinguish between ”*Spontaneous*” and ”*Synchronized*” behaviours, it is much more complex to distinguish between ”Spontaneous” and ”Alone” movements. This is mainly because a synchronized movement is, by definition, synchronized. If both hands tend to be in physical alignment, this implies a *Synchronized*” state. On the other hand, when people are instructed to move freely (autonomous behavior), their respective movements are not aligned in any sense. Nevertheless, since ”spontaneous synchronization” occasionally emerges, one can detect it using ML algorithms.

### Model’s input

As explained above, one can easily detect different modes given enough observation time. However, the model’s input is a **single snap-shot** containing both hands position and velocity vectors. Another way to look at it is that we try to deduce the synchronization state based on a single picture of the hands (e.g., try to guess the synchronization state depicted in Fig. 2) plus meta-data containing their respective velocities in all axes.

**Figure 3:**
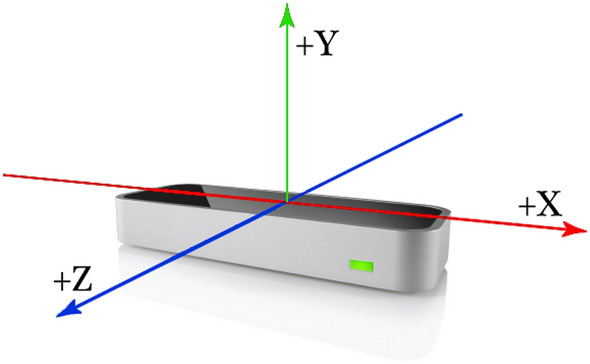
The leap motion controller X–Y–Z orientation.

As shown in Fig. 3, the *X* axis represents the participants’ horizontal position with respect to the controller. The *Y* axis indicates the participants’ vertical (height) distance from the LMC. These X-Y-Z distances are reported in absolute values (mm) from the LMC center to the hand (palm center).

**Figure 4:**
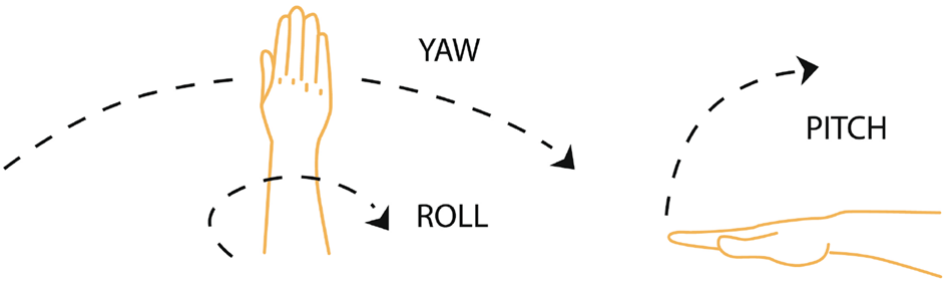
Hand’s different attitudes.

The LMC device can both track and record the following data:

1. 3*D* position vector of each hand (measured between the device’s center and the palm’s center).
2. 3*D* velocity vector of each hand (measured in $\frac{mm}{s}$).
3. The orientation of each hand—yaw, pitch, and roll (demonstrated in Fig. 4).
4. 3*D* position vector of the elbow and the wrist of each hand.
5. a ’grab’ metric. The grab indicates how close a hand is to be a fist—any fingers that aren’t curled will reduce grab strength.

All the above features are summed to 13 figures for each hand—four 3*d* vectors (12) + a grab measure(1).

We are dealing with tabular data. Thus, one of the most efficient *ML* algorithms to apply is XGBoost^43^.

### Machine leaning algorithms

In the context of this study, the choice of a specific machine learning algorithm is less important than its use as a tool to help test the main hypothesis—that different mental synchronization can be distinguished. In other words, distinguishing between these states is relatively easy given sufficient data and time. However, if such classification is possible based on a **single** hands snapshot (and section "Model evaluation" shows it does), it raises the question: why? What does the algorithm ’*see*’ in a single snapshot that distinguishes one mental state from the other? We address this very question in section "Discussion".

The following classification algorithms were tested:

- Support Vector Machine (SVM)
- XGBoost
- Naive Bayes
- A 3-layer Dense Neural Network
- Random Forest

Out of all the algorithms the best results were achieved with XGBoost.

#### XGBoost

XGBoost—Extreme Gradient Boosting^44^, is a scalable, distributed gradient-boosted decision tree machine learning library. It can be used for supervised learning classification tasks. Supervised learning algorithms find patterns in a labeled data set according to a set of features in the data and accordingly can classify new data according to the same type of features. In a nutshell, decision trees create a model that predicts the label by evaluating a tree of questions regarding the features. Gradient Boosting Decision Trees (*GBDT*) create a model consisting of an *ensemble* of multiple decision trees. The algorithm iteratively trains multiple shallow decision trees, using the error of the previous iteration to fit the next iteration. Finally, a classification is done based on a weighted sum of all the shallow decision trees. As stated above, XGBoost is considered to be the prominent algorithm in tabular supervised learning^43^. This scalable, distributed gradient-boosted decision tree machine learning library is utilized for its effectiveness in supervised learning classification tasks. The algorithm finds patterns in a labeled dataset based on features and can classify new data using the same feature set. At its core, decision trees create a model that predicts labels through a series of feature-based queries. Gradient Boosting Decision Trees (GBDT), and in our case XGBoost, extend this by constructing an ensemble of multiple decision trees. The algorithm iteratively trains numerous shallow decision trees, leveraging the error of the preceding iteration to inform the subsequent one. The final classification is determined based on a weighted sum of all the shallow decision trees. It’s worth noting that XGBoost has been recognized as a leading algorithm in tabular supervised learning^43^.

### Train-test split

12 different volunteers participated in the mirror game. Each person behaves differently and understands differently the ”Be synchronized” task. Since the classifier objective is not to study the synchronization patterns of a specific person but to learn the underlying patterns common among all participants, a subject-independent classifier is mandatory. Thus, all algorithms were trained using 9 participants while tested with 3. In other words, the algorithms couldn’t learn their specific pattern and had to rely on the common pattern found in the first 9 subjects in the training phase.

## Results

In the next two sections, we address the two questions proposed in Section "Our mirror game experiment"—can one distinguish between the different states based on a single frame of the mirror game, and what insights emerge from the data itself? We start with the first question.

### Model evaluation

As stated in Section "Machine leaning algorithms", several algorithms were tested. One can see their relative performances in Table 1.

**Table 1:** Comparison of different algorithms used in the study, sorted by F1-Score.

Algorithm	Accuracy	F1-Score	Precision	Recall
XGBoost	0.88	0.87	0.9	0.87
Random Forest (500 trees)	0.876	0.873	0.903	0.876
SVM	0.868	0.866	0.885	0.868
Neural Nets (3 layers)	0.865	0.863	0.883	0.865
Naive Bayes	0.842	0.842	0.844	0.843

The XGBoost algorithm achieved the highest score, both on accuracy and *F*1 score.

We trained multiple XGBoost models with different hyper-parameter configurations on the data. Table 2 presents these models and their respective hyper-parameters and performance metrics, including run-time, accuracy, precision, recall, and F1 score. For all models, the ”booster” hyper-parameter was set to ”*gbtree*” since other settings resulted in significantly lower performance (by approximately $10\%$).

**Table 2:** Results of the trained model with different hyper-parameters.

Learning rate	# Estimators	Max depth	Min child weight	Max delta step	Time taken (secs)	Accuracy	Avg. Precision	Avg. Recall	Avg. F1 Score
0.5	4000	6	1	0	30.37	0.8795	0.9	0.87	0.87
0.2	8000	6	1	0	66.65	0.88	0.9	0.87	0.87
0.5	4000	10	1	0	29.55	0.8712	0.88	0.87	0.86
0.7	2000	6	1	0	16.66	0.8751	0.89	0.87	0.87
0.9	2000	15	1	0	15.03	0.8722	0.89	0.87	0.87
0.5	4000	15	1	0	29.93	0.8679	0.88	0.86	0.86
0.5	4000	6	5	0	27.5	0.8799	0.87	0.87	0.87
0.5	4000	6	10	10	25.5	0.8814	0.89	0.88	0.87
0.5	4000	6	2	2	28.07	0.8756	0.89	0.87	0.87
0.5	8000	6	1	0	50.01	0.8792	0.89	0.87	0.87
0.5	100	6	1	0	3.04	0.8	0.79	0.82	0.8

**Figure 5:**
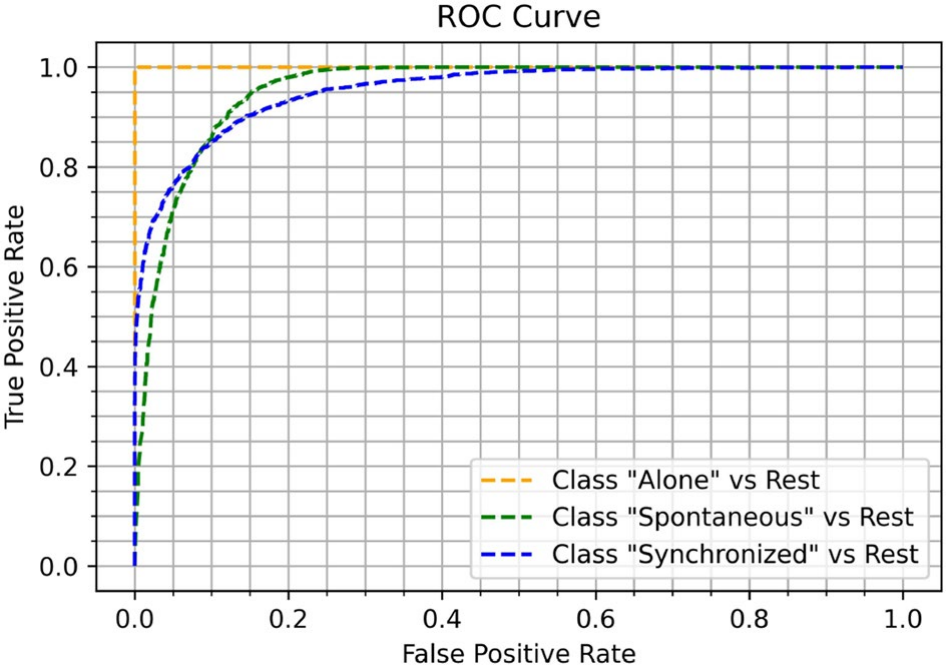
Roc curve.

**Figure 6:**
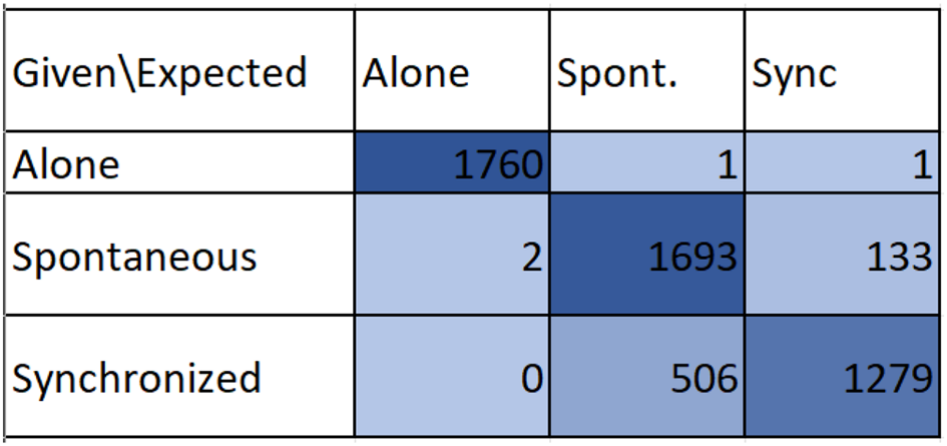
Model accuracy confusion matrix.

We chose to use the first classifier because it offered the optimal balance between accuracy metrics and training time. The classifier’s accuracy, as shown in the table above, is $87.9\%$ (precision—$90\%$, recall—$87\%$, F1 score—$87\%$), meaning the classifier can distinguish (with high certainty) between the different states. One can see the *ROC* Curve in Fig. 5 and the full confusion matrix in Fig. 6.

What can be learned from the above matrix? First, the ‘Alone’ mode is much easier to distinguish from the other modes. Second, there is an inherent difference between spontaneous and intentional synchrony modes based on their type I/II errors. Given the real state is spontaneous movement (second row in Fig. 6), the algorithm error rate is $\approx 7\%$. However, given the real state is intentional synchrony, the error rate is four times higher ($\approx 28\%$). Why is it so? After inspecting the experiments and the data, the reason for this phenomenon is as follows: misclassifying a spontaneous state as a sync state can occasionally happen if both hands are accidentally aligned (remember that we only use a single snapshot). It can happen (since both hands move spontaneously) but is uncommon. However, when the subject is in an intentional synchrony state, to ‘sync’, one needs to *compensate* if the hands are not aligned. The positional compensation process, by its nature, causes changes in the velocity (one needs to change their hand’s velocity to maintain the same position), causing the system to ‘see’ these moments as spontaneous movements. Put simply, when people think of ”synchronization,” they usually focus on being in the same place rather than moving at the same speed. But can the results be expanded beyond a simple accuracy figure? What can be learned from all the gathered data? How do people perceive and understand the task: ”Be synchronized” and what are the behavioral manifestations of this understanding?

But can the results be expanded beyond a simple accuracy figure? What can be learned from all the gathered data? How do people perceive and understand the task: ”Be synchronized” and what are the behavioral manifestations of this understanding?

## Discussion

In the following section, we present the behavioral manifestations of the different synchronization modes. Let us start with the velocity.

### Velocity

**Figure 7:**
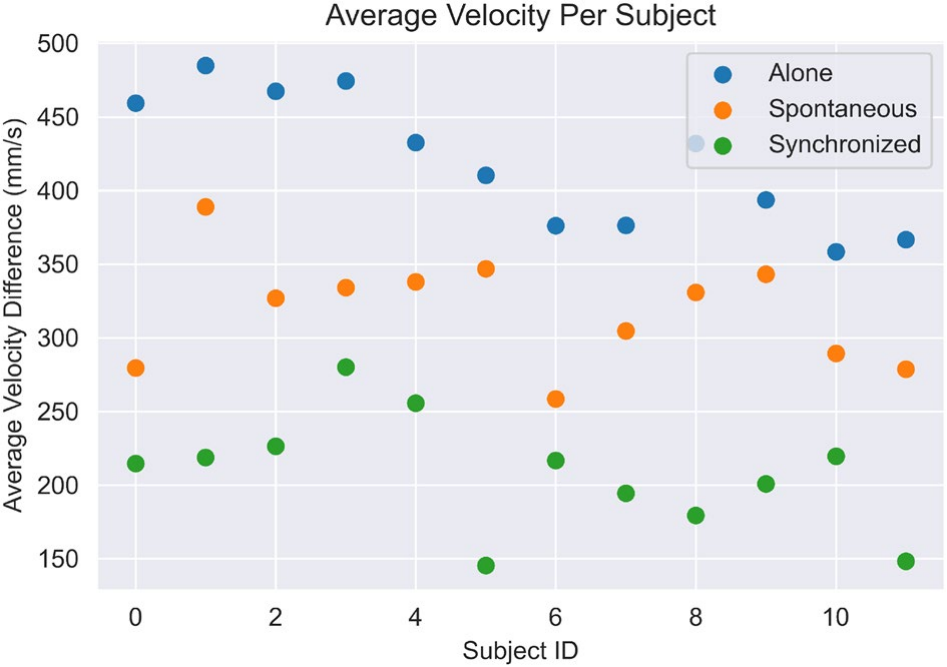
The average velocity difference of each subject in each of the 3 tests. The difference can be clearly seen for each of them. Blue: Alone; Orange: Spontaneous; Green: synchronized.

As can be clearly seen from the data, people tend to move at different velocities in different scenarios. Apparently, this could be seen as a trivial observation. Still, the data shows consistent behavior across all subjects—the highest velocity is reached in ‘Alone’ mode, a medium velocity is reached in ‘Spontaneous’ mode, and the lowest velocity was recorded for all subjects in ’ Synchronization’ mode. The data can be depicted in Fig. 7. These findings are consistent with the work of Noy et al.^45^, which used a whole-body mirror game task and showed that slower movements lead to higher synchrony. However, our findings contradict an earlier study by Noy et el^11^. In that earlier study, the researchers used one-dimensional mirror game tasks and found that faster movements are associated with a higher level of synchrony. The discrepancy between these findings may stem from the level of cognitive load that an individual experiences. More specifically, creating synchronized motion together by moving handles in the one-dimensional mirror game may be less consuming and require lower cognitive load than synchronizing with the whole body or parts of it (as in the current study). We, therefore, conclude that our findings support the idea that the relationship between velocity and synchrony depends on the level of cognitive load that is required of the task, with faster movements leading to higher synchrony in a task that requires a lower cognitive load, but slower movements leading to higher synchrony in a task that requires high cognitive load^45^.

**Figure 8:**
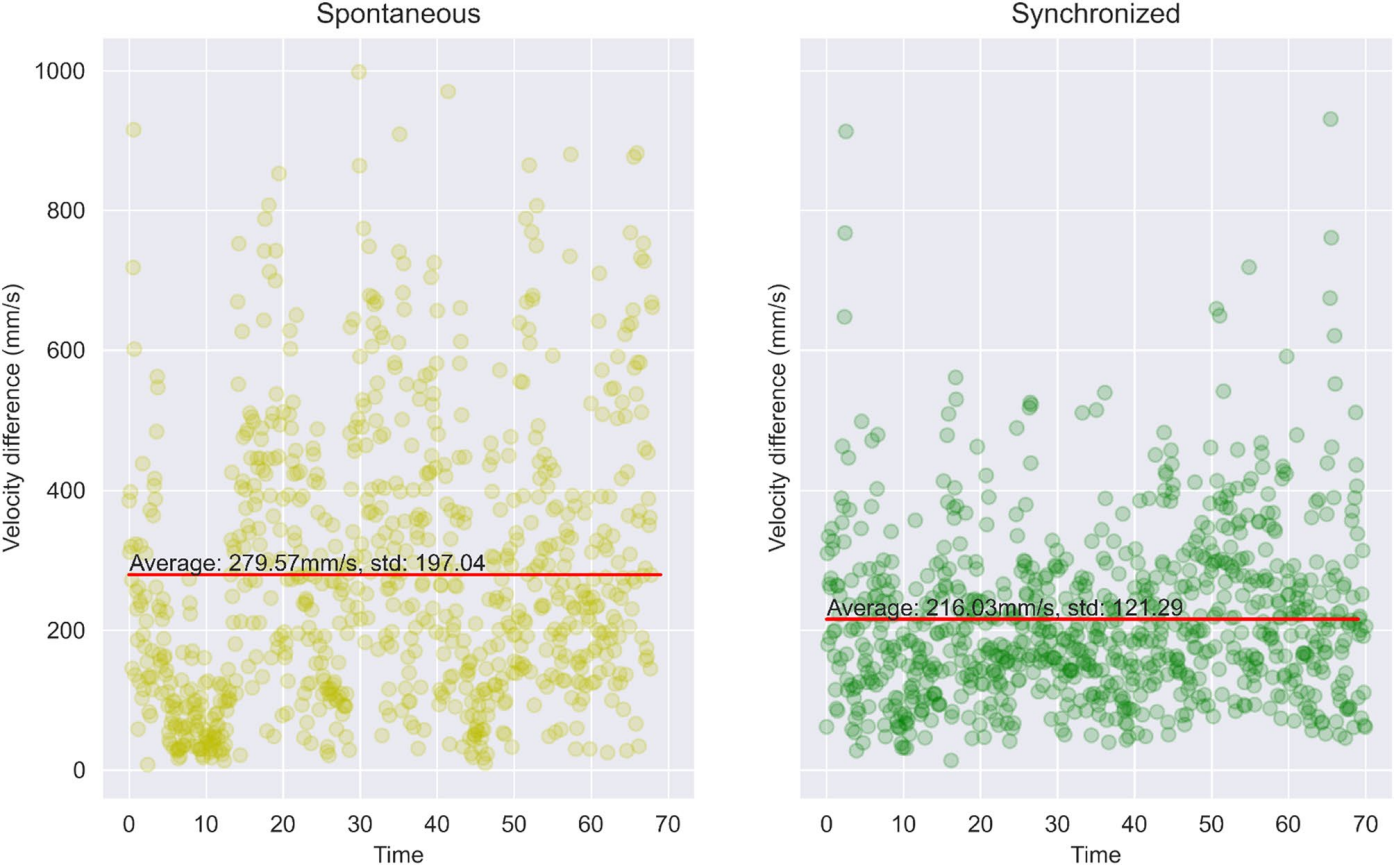
The difference in velocities between the two subjects as time passes for a single subject pair (which is symbolic of the whole group).

### Position

**Figure 9:**
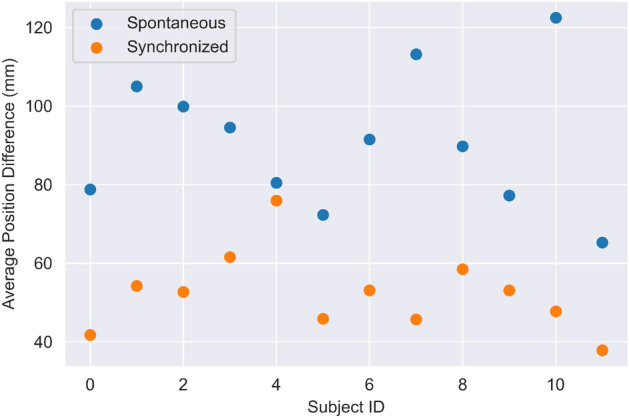
The average hands’ distance in Spontaneous and IS modes. One can see that people tend to be closer in synchronization mode.

What can we say regarding the hands’ distance over time? Almost overall, the distance between participants (as measured in *mm* between the two hands) is greater in spontaneous mode than in IS mode. The results are depicted in Fig. 9.

Among *all* participants ($x-axis$) one can see that the average distance in IS mode is smaller than the Spontaneous mode. For some (e.g., *ID* 1,9) the gap is significant while for others (e.g., *ID* 4,11) the gap is relatively small. However, being closer in IS mode is a common train across all participants. One can see that synchronization is also interpreted as ”being closer to one another”.

This, in addition to the subject’s average movement speed being much greater (Fig. 8)—again, most likely due to not having to coordinate movements with another person (consciously or subconsciously). Another important conclusion one can draw from the data is that a subject’s view in synchronization is dependent to a very high extent on the height (the $y-axis$) of the hands. Thus, people in an intentional synchrony state, tend to correlate their respective *y* position values (vertical) more than the other axes.

#### Sine wave behaviour

**Figure 10:**
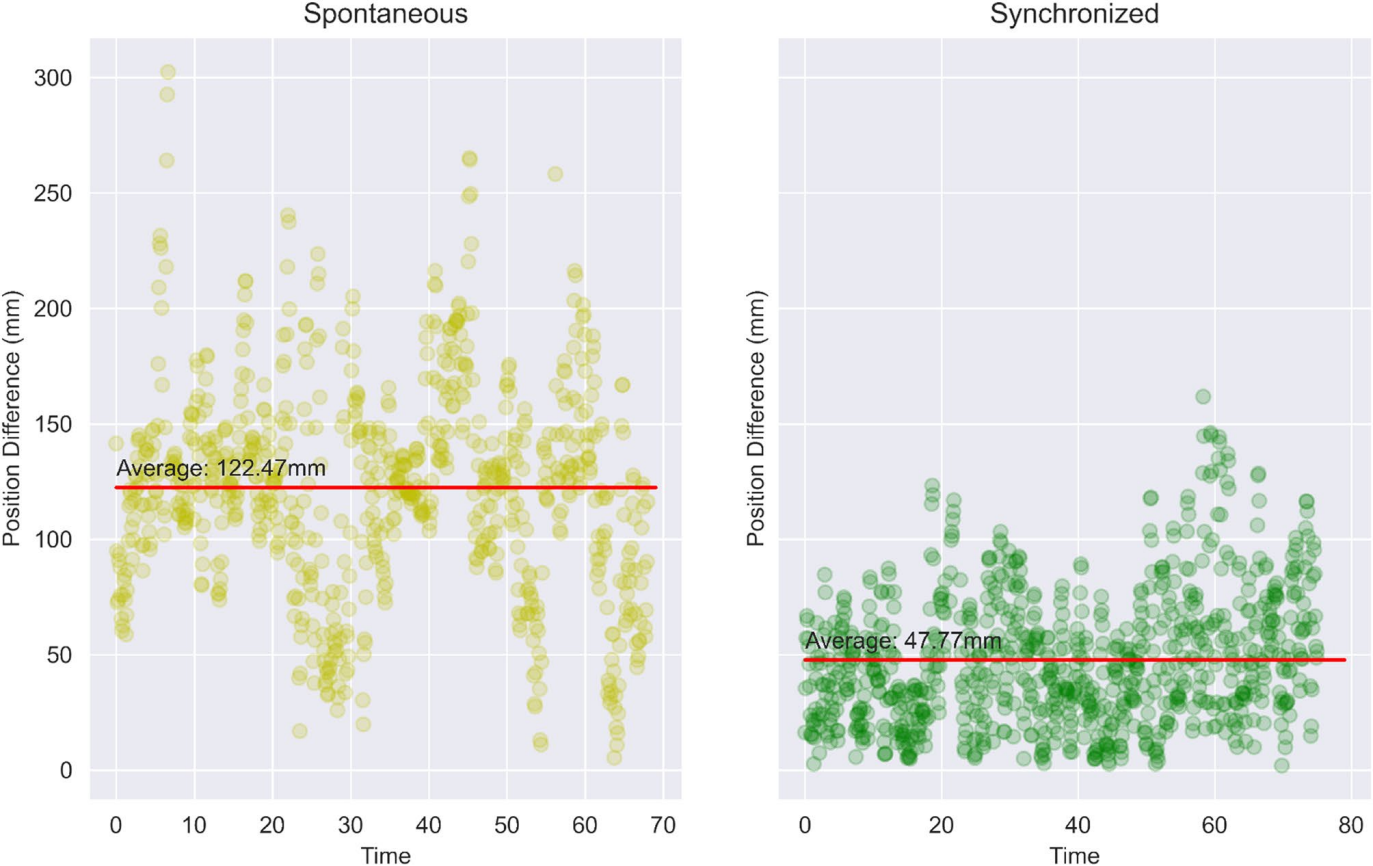
Position differences over time between two subjects as time passes for a single subject pair (which is symbolic of the whole group).

Figure 9 encapsulates an entire 70 *sec* session to a singular point—average. Figure 10 depicts the entire session. In the figure, one can see the position difference (same as the $y-axis$ in Fig. 9) over the span of the entire session - 70 *sec*. The figure shows the data of one subject but the pattern shown was common among all participants.

Interestingly, one can notice a slight sine amplitude tendency in all positional differences of the Synchronized and Spontaneous states which could indicate that a subject, every time after furthering from the other subject, feels obligated in some way to come closer to it again after a period of time. This pattern is in line with previous findings that showed that during the mirror game, participants showed a pattern of getting in and out of synchronization^46–48^. It has been suggested this pattern of falling out of synchronization allows participants to increase the complexity and novelty of the interaction. Within mutual adaptation between participants, when they feel confident, they are willing to temporarily reduce synchronization to make the interaction more interesting and meaningful by increasing its complexity, and following this change, they again manage to restore the synchronization. This occurs spontaneously and repeatedly along the interaction^48^.

IS is linked to prosocial behaviours^5,49^ and reduced IS was found to be associated with deficits in social cognition, e.g., in schizophrenia^50–53^, in ASD^54,55^ and in ADHD^56^. Classifying patterns of IS during real-life interaction may pave the way for future studies to develop interventions for disorders such as ASD. By precisely recognizing patterns of interpersonal synchrony during actual interactions, we can use the algorithm to provide real-time feedback for successful IS. Several existing Interventions for ASD aim at improving interpersonal synchronization. Such interventions include Music Therapy which includes rhythmic synchronization activities, such as playing musical instruments or engaging in group singing. Dance/Movement Therapy. Joint Attention Intervention that contains activities that encourage eye contact, turn-taking, and shared focus on objects or events^57–59^. Algorithms for assessing interpersonal synchrony can play a valuable role in interventions aimed at improving interpersonal synchronization. Real-time feedback is essential for individuals with ASD to understand and adjust their behavior during social interactions. Algorithms can be integrated into interactive systems or wearable devices to provide immediate feedback on synchrony levels. This feedback can guide individuals to synchronize their movements, gestures, or vocalizations with their interaction partners, facilitating real-time adjustments and promoting improved synchrony. Moreover, by analyzing the collected data, therapists can gain insights into the specific areas of synchrony that individuals with ASD struggle with. This information may help in developing personalized intervention plans that target the specific areas needing improvement paving the way for individualized Intervention planning. Using real-time classification algorithms may allow the development of adaptive interventions. Such interventions may adjust in real time based on the individual’s progress and needs. By continuously monitoring interpersonal synchrony, algorithms can provide immediate feedback and dynamically modify the intervention activities to optimize learning and engagement. This adaptability ensures that interventions remain tailored and responsive to the individual’s unique requirements.

## Conclusions and future work

This work offered a data-driven method for understanding different synchronization states. Unlike previous methods that measure IS as a time series, we only inspect a single snapshot and deduce from it the corresponding state (Spontaneous or IS modes). An XGBoost ML model reached almost $90\%$ accuracy between Spontaneous and IS modes. In addition, real experiments conducted with a dozen participants confirm the validity of these findings. Up till now, the literature on IS has mainly focused on developing measurements for IS^11,60^ as well as tasks that measure spontaneous synchrony or intentional synchrony^55,56^. There has been little study focused on the idea of constructing an algorithm that can identify interpersonal synchrony. Such an algorithm may have important implications for the development of new metrics for assessing different types of synchrony protocols of real-time human social interactions. Importantly assessing IS in real-time can pave the way for developing adaptive treatment interventions with real-time feedback. Hence, developing an algorithm that classifies patterns of IS during real-life interaction may have implications for understanding social interaction and diagnosing and developing treatment strategies for social deficits associated with conditions such as ASD.

The focus of the current work is hand postures using the in-depth camera, It will be interesting to see whether the patterns of velocity and interpersonal space also take place for the whole body movement. Whole-body synchrony refers to movements that are often subtle and require the coordination of multiple body parts to achieve synchronization. During our everyday social interaction, we tend to coordinate not only our hand movements with those of others but also our head movements as well as other parts of the body. Hence, measuring whole-body synchrony provides a more accurate and comprehensive understanding of the coordination that occurs between individuals during social interactions. Indeed, whole-body synchrony was significantly related to the therapeutic alliance between the therapist and patient, demonstrating the importance of measuring whole-body synchrony in clinical contexts^61^. Finally, it has been suggested that there may be different head- versus body synchrony effects. More specifically, synchronized head movement was found to be associated particularly with the macro-outcome of psychotherapies (global therapy success), whereas synchronized body movement predicted short-term micro-outcome at the session level^62^.

### Future work

One limitation of the current study is that it did not examine the influence of nationality, culture, gender, and age. Previous studies have consistently shown that individuals synchronize more with in-group members who share characteristics like nationality and gender while demonstrating less synchronization with out-group members^63^. Recent research has revealed differences in the degree of synchronization between in-group dyads (consisting of persons from the same nationality group) and inter-group dyads (consisting of individuals from other nationality groups)^55,56,64^. These findings highlight people’s natural tendency to associate themselves with individuals who are similar to them, resulting in increased synchrony^65^.

We call for future studies to examine the impact of these parameters examine the impact of these factors.
